# CRISPR-Mediated Metabolic Engineering of *Escherichia coli* W for Selective Biopurification of Stachyose from Soybean Molasses

**DOI:** 10.3390/microorganisms14051029

**Published:** 2026-05-01

**Authors:** Haotian Wang, Guoyu Liu, Jia Liu, Yifei Zhu, Jingmei Huang, Shiwei Liu, Huaping Pan, Yafang Li, Yan Zou, Xueying Zeng, Guankai Hao, Haizhi Li, Shufan Yang, Shenglin Duan, Juxiu Li, Peng Yuan

**Affiliations:** 1 College of Food Science and Engineering, Northwest A&F University, Yangling 712100, China; wht1275930132@126.com (H.W.); yifei_zhu993@163.com (Y.Z.); 2 Key Laboratory of Foods for Special Medical Purposes for Diabetes, China National Research Institute of Food and Fermentation Industries, Beijing 100015, China; lgy17091037820@163.com (G.L.); liujiajessica@163.com (J.L.); myxi0811@163.com (J.H.); 15101156761@163.com (S.L.); 15808023960@163.com (Y.Z.); m17278100584@163.com (X.Z.); 15035618920@139.com (G.H.); li_haizhi@163.com (H.L.); bunnyfunk@outlook.com (S.Y.); dslbeijing@163.com (S.D.); 3 School of Pharmacy, Jiangxi University of Chinese Medicine, Nanchang 330004, China; huaping@jxutcm.edu.cn (H.P.); 20201009@jxutcm.edu.cn (Y.L.)

**Keywords:** stachyose, soybean molasses, biopurification, CRISPR–Cas9, *Escherichia coli* W

## Abstract

Soybean molasses, a by-product of alcohol-based soy protein concentrate production, is rich in stachyose and other functional oligosaccharides, but its high sucrose content and other fermentable non-target sugars hinder the efficient purification of stachyose. In this study, the sugar-utilization patterns of four commonly used microbial chassis or production strains, *Escherichia coli* W, *E. coli* BL21, *Saccharomyces pastorianus* Weihenstephan 34/70, and *Komagataella phaffii* (formerly *Pichia pastoris*) GS115, were systematically compared to identify a suitable host for selective stachyose enrichment. Among them, *E. coli* W showed the best performance in rapidly consuming non-target sugars while retaining stachyose. Based on this strain, a CRISPR–Cas9 engineering strategy was applied by deleting the endogenous α-galactosidase gene *melA* and overexpressing the sucrose permease gene *cscB*. The resulting strain selectively and nearly completely removed sucrose and other non-target sugars from soybean molasses, increasing the proportion of stachyose from <30% to >90% of total soluble solids. Further optimization of nitrogen source level, inoculum size, and initial °Brix improved fermentation performance. These results demonstrate an effective biological pre-purification strategy for selective stachyose enrichment from soybean molasses.

## 1. Introduction

Stachyose is a raffinose family oligosaccharide (RFO) composed of two galactose units, one glucose unit, and one fructose unit. It is widely distributed in leguminous plants and members of the Lamiaceae family [1,2,3,4]. In recent years, accumulating evidence has shown that nondigestible RFOs, including stachyose, can modulate gut microbiota composition, enhance host immune function, and improve glucose and lipid metabolism, highlighting their promising prebiotic and metabolic regulatory potential [2,5,6,7]. These advances have substantially expanded the application potential of stachyose in foods, nutraceuticals and precision nutrition strategies [4,7].

Soybean molasses is a major co-product generated during the ethanol extraction process used for soy protein concentrate production [8] and it is increasingly regarded as a sustainable feedstock for industrial biotechnology [9]. On a dry matter basis, its carbohydrate content typically reaches 50–60%, with sucrose, raffinose and stachyose as the predominant sugar components [3,10]. It also contains appreciable amounts of glucose, fructose, proteins, saponins, isoflavones, ash and other phytochemicals, forming a complex matrix [9]. Because stachyose can account for a considerable proportion of total soluble sugars in some soybean molasses preparations (approximately 30%), soybean molasses has been regarded as a cost-effective and sustainable feedstock for stachyose production [3,9].

However, the coexistence of stachyose with various mono- and disaccharides in soybean molasses greatly increases the difficulty of downstream separation and purification [11]. The close similarity in molecular weight and hydrophilicity between sucrose and stachyose markedly reduces the selectivity of membrane processes and conventional chromatographic methods [12], while fouling caused by complex non-sugar impurities further elevates energy consumption and maintenance costs [3]. Consequently, it remains difficult to achieve economically viable production of high-purity stachyose from soybean molasses using traditional physical separation methods alone.

Biopurification, defined here as the selective removal of fermentable sugars by microorganisms under mild and controllable conditions while retaining target oligosaccharides, has emerged as a promising strategy for the valorization of soybean by-products [9,11,12]. Previous studies have shown that wild-type yeasts, molds, and lactic acid bacteria can preferentially metabolize glucose, fructose, and sucrose in soybean-derived substrates, thereby enabling the initial enrichment of raffinose and stachyose in the residual sugar fraction [11,13]. Nevertheless, this strategy still faces several inherent limitations. Maintaining a high sucrose depletion rate often requires prolonged fermentation or mixed-culture systems, making process operation and control more complex [14]. More critically, although stachyose can be enriched in the early fermentation phase, it is frequently subjected to progressive degradation in the middle and late stages, which compromises product recovery and final purity [11,12]. At the mechanistic level, many microorganisms harbor endogenous glycosidase systems, such as α-galactosidases, that non-selectively hydrolyze RFOs during extended cultivation [15]. In parallel, reinforcement of sucrose metabolism is often accompanied by nonspecific hydrolysis of oligosaccharides by invertases or related sucrose-hydrolyzing enzymes, further destabilizing the target oligosaccharide fraction [16]. Thus, current biopurification systems face a fundamental trade-off between efficient removal of unwanted sugars and effective retention of stachyose, and this remains a key technical bottleneck for highly selective enrichment [12].

To address these challenges, an ideal chassis microorganism should possess well-defined and readily tunable sugar metabolic pathways that enable preferential consumption of sucrose and monosaccharides while minimizing stachyose degradation. In this context, we systematically compared the sugar-metabolic characteristics of *Escherichia coli* W [16,17,18], *E. coli* BL21 [19,20], lager-brewing *Saccharomyces pastorianus* W34/70 (hereafter W34/70), [21] and *Komagataella phaffii* (formerly *Pichia pastoris*) GS115 (hereafter GS115) [22,23] in soybean molasses. Among the tested strains, *E. coli* W showed clear advantages in fermentation rate, substrate adaptability, and amenability to metabolic engineering, consistent with recent reports supporting its potential as a robust chassis for sucrose- and molasses-based bioprocesses [24].

Compared with conventional bacterial genome engineering approaches, CRISPR/Cas9-based editing offers advantages in programmability, editing efficiency, and the ability to perform precise and potentially multiplex chromosomal modifications, making it particularly suitable for pathway-level metabolic engineering in bacterial hosts [25]. Based on this result, we employed a CRISPR–Cas9 genome editing system to delete the endogenous α-galactosidase gene *melA* in *E. coli* W, thereby blocking a major route of stachyose hydrolysis [26,27,28,29,30]. In parallel, we overexpressed the sucrose permease gene *cscB* to enhance sucrose uptake and increase flux through the sucrose utilization pathway [31,32]. Subsequently, key process parameters, including inoculum size, initial sugar concentration, pH, temperature and agitation, were systematically optimized to construct a biopurification system capable of rapid sucrose depletion coupled with efficient stachyose preservation [11].

In this study, we developed an efficient biopurification strategy for selective stachyose enrichment from soybean molasses. By integrating chassis selection, metabolic engineering, and process optimization, this work provides a practical framework for the selective enrichment of stachyose from soybean molasses. The results showed that *E. coli* W was the most suitable chassis among the strains tested, and that rational engineering of sucrose uptake and stachyose hydrolysis could substantially improve the selectivity of soybean molasses biopurification.

## 2. Materials and Methods

### 2.1. Reagents and Kits

Unless otherwise specified, all chemical reagents were purchased from Sangon Biotech (Shanghai, China) or Sigma-Aldrich (St. Louis, MO, USA). Culture media, buffers, and antibiotics were obtained from Sangon Biotech (Shanghai, China). PCR amplifications were performed using 2× Rapid Taq Master Mix (Vazyme, Nanjing, China). One-step cloning was carried out with the ClonExpress^®^ Ultra One Step Cloning Kit (C115, Vazyme, Nanjing, China). Plasmid DNA was extracted using the FastPure^®^ Plasmid Mini Kit (DC201, Vazyme, Nanjing, China), and DNA fragments were recovered from agarose gels using the FastPure^®^ Gel DNA Extraction Mini Kit (DC301, Vazyme). The pEASY^®^-T3 Cloning Kit and Trans DNA Marker II were purchased from TransGen Biotech (TransGen Biotech, Beijing, China).

### 2.2. Plasmids and Strains

#### 2.2.1. Strains and Plasmids

The strains and plasmids used in this study are listed in Table 1. *Escherichia coli* DH5α was used as the cloning host for plasmid propagation. *E. coli* W (ATCC 9637), *E. coli* BL21, *Komagataella phaffii* GS115, and *Saccharomyces pastorianus* Weihenstephan 34/70 (W34/70) were used as comparator strains from the laboratory collection. *E. coli* W served as the parental chassis strain in this study, whereas *E. coli* BL21 is a laboratory expression strain, GS115 is a laboratory *K. phaffii* host strain, and W34/70 is an industrial lager-brewing strain. The engineered strains *E. coli* W *melA*^−^, in which the chromosomal *melA* gene was deleted, and *E. coli* W *melA*^−^ *cscB*^+^, in which a J23100–*cscB* expression cassette was integrated at the *melA* locus, were constructed in this study.

#### 2.2.2. Plasmid Construction

(1) Construction of pEASY-T3-sgRNA-*melA*

The *melA*-targeting sgRNA cassette (sgRNA-*melA*) was amplified by PCR from plasmid pEASY-T3-sgRNA using primers F-gRNA-*melA* and R-gRNA-*melA*. The PCR product was purified from agarose gels, A-tailed with Taq DNA polymerase at 72 °C for 10 min, and ligated into the linearized pEASY-T3 vector (Amp^R^) using the pEASY^®^-T3 Cloning Kit (TransGen Biotech, Beijing, China) according to the manufacturer’s instructions. The ligation mixture was transformed into *E. coli* DH5α competent cells, and transformants were selected on LB agar plates containing ampicillin. Positive clones were identified by Sanger sequencing (Ruibobio, Beijing, China). The verified construct was designated pEASY-T3-sgRNA-*melA*.

(2) Construction of pEASY-T3-sgRNA-*melA*-*melA^−^*

For construction of the *melA* deletion plasmid, the upstream and downstream homology arms of *melA* were amplified by PCR from the genomic DNA of *E. coli* W (ATCC 9637) using primer sets F-*melA*-T3-up/R-*melA*-down-up and F-*melA*-up-down/R-*melA*-T3-down, respectively. The PCR products were recovered from agarose gels. Plasmid pEASY-T3-sgRNA-*melA* was linearized by inverse PCR using primers F-down-T3-loop and R-up-T3-loop, and the amplification product was gel-purified. The purified *melA* homology arm fragments were assembled into the linearized pEASY-T3-sgRNA-*melA* backbone by one-step cloning using the ClonExpress^®^ Ultra One Step Cloning Kit (C115, Vazyme, Nanjing, China). The cloning mixture was transformed into *E. coli* DH5α competent cells, and transformants were selected on LB agar plates supplemented with ampicillin. Positive clones were confirmed by Sanger sequencing (Ruibobio, Beijing, China), and the resulting plasmid was designated pEASY-T3-sgRNA-*melA*-*melA*^−^.

(3) Construction of plasmid pEASY-T3-sgRNA-*melA*-J23100-*cscB*

Genomic DNA of *E. coli* W (ATCC 9637) was used as the template to amplify the *cscB* coding sequence using primers F-J23100-*cscB* and R-down-*cscB*-arm (primer sequences are listed in Table 2). The J23100–*cscB* expression cassette was subsequently generated by overlap extension PCR using primer F-uparm-J23100-arm. Plasmid pEASY-T3-sgRNA-*melA* was used as the backbone, and the original upstream and downstream *melA* homology arms were retained. The J23100–*cscB* expression cassette was inserted between the two homology arms at the multiple cloning site to generate the integrative editing plasmid pEASY-T3-sgRNA-*melA*-J23100-*cscB*. The assembly reaction was performed using the ClonExpress^®^ Ultra One Step Cloning Kit (C115, Vazyme, Nanjing, China) according to the manufacturer’s instructions. The ligation mixture was transformed into *E. coli* DH5α, spread on LB agar plates containing ampicillin, and incubated at 37 °C for 14 h. Single colonies were picked for plasmid extraction, and the presence of the correct J23100–*cscB* expression cassette and *melA* homology arm sequences was verified by restriction analysis and Sanger sequencing (Ruibobio, Beijing, China). The confirmed construct pEASY-T3-sgRNA-*melA*-J23100-*cscB* was used as the CRISPR–Cas9 editing vector for integration of the J23100–*cscB* cassette into the *melA* locus of *E. coli* W.

All primers used in this study are listed in Table 2.

### 2.3. Molecular Biology Methods

#### 2.3.1. PCR Amplification of DNA Fragments

Plasmid DNA or single colonies were used as templates for PCR amplification. All primers were synthesized by Ruibobio (Beijing, China). Target gene fragments were amplified using 2× Rapid Taq Master Mix (Vazyme, Nanjing, China). The PCR program was as follows: initial denaturation at 95 °C for 3 min; 28 cycles of 95 °C for 20 s, 56 °C for 20 s, and 72 °C for 1–4 min (the extension time was adjusted according to an elongation rate of 1.5 kb/min for the 2× Rapid Taq Master Mix); and a final extension at 72 °C for 2 min, followed by holding at 4 °C.

The detailed compositions of the 50 μL and 20 μL PCR reaction mixtures are provided in Appendix A (Table A1 and Table A2).

#### 2.3.2. Agarose Gel Electrophoresis and Purification of PCR Products

PCR products were analyzed by electrophoresis on 1% (*w*/*v*) agarose gels prepared in 1× TAE buffer and stained with 4S Green nucleic acid stain.

PCR products were separated in 1× TAE buffer at 120 V for 30 min and visualized using a blue-light transilluminator (470 nm). Target fragments were excised from the gel and purified using the FastPure^®^ Gel DNA Extraction Mini Kit (DC301, Vazyme, Nanjing, China) according to the manufacturer’s instructions.

#### 2.3.3. One-Step Cloning

Homologous DNA fragments were assembled into linearized vectors using the ClonExpress^®^ Ultra One Step Cloning Kit (C115, Vazyme, Nanjing, China) according to the manufacturer’s instructions.

#### 2.3.4. Plasmid Extraction from *E. coli* DH5α

Plasmid DNA was extracted from *E. coli* DH5α using the FastPure^®^ Plasmid Mini Kit (DC201, Vazyme, Nanjing, China) following the manufacturer’s protocol.

#### 2.3.5. Restriction Digestion

Restriction digestion of plasmid DNA was carried out using restriction endonucleases (Takara Bio, Kusatsu, Shiga, Japan) according to the manufacturer’s recommendations.

#### 2.3.6. Preparation and Transformation of Chemically Competent *E. coli* Cells

Chemically competent *E. coli* cells were prepared using a Super Competent Cell Preparation Kit (Sangon Biotech, Shanghai, China) according to the manufacturer’s instructions. Frozen competent cells stored at −80 °C were thawed on ice for 15 min. Ligation mixtures or plasmid DNA were added to the competent cells and gently mixed, followed by incubation on ice for 20 min. Cells were then heat-shocked at 42 °C for 80 s and immediately returned to ice for 3 min. The transformation mixtures were spread onto LB agar plates containing the appropriate antibiotics and incubated at 37 °C for 14 h.

#### 2.3.7. CRISPR–Cas9-Mediated Genome Editing

In this study, plasmid pRed_cas9_recA_Δpoxb300 (hereafter referred to as the 300 plasmid; Kan^R^, temperature-sensitive replication at 30 °C) and pEASY-T3-based editing vectors were used to construct a CRISPR–Cas9 genome editing system for *melA* deletion and J23100–*cscB* cassette integration in *E. coli* W.

The gRNA spacer targeting *melA* was designed based on the *melA* coding sequence according to the canonical 5′-N_20_-NGG rule. Multiple potential target sites located in the 5′, central, and 3′ regions of the *melA* coding sequence were screened. Spacer candidates with a GC content of 40–60%, without long homopolymeric tracts or strong self-complementarity, and with unique matches in the *E. coli* W genome were preferentially selected. Each gRNA cassette contained the J23119 promoter, a 20 bp spacer, the gRNA scaffold, and a terminator, and was amplified as a single fragment using a pair of 80 bp primers (e.g., F-gRNA-*melA*/R-gRNA-*melA*; Table 2).

The gRNA fragment was amplified by PCR using 2× Rapid Taq Master Mix, separated on a 1% agarose gel, and purified as described above. The purified fragment was A-tailed with Taq DNA polymerase at 72 °C for 10 min and ligated into the linearized pEASY-T3 vector to construct pEASY-T3-sgRNA-*melA*.

To improve editing efficiency, the upstream and downstream homology arms (~500 bp each) of *melA* and, when required, the J23100–*cscB* expression cassette were inserted into the multiple cloning site of pEASY-T3-sgRNA-*melA* so that both the gRNA and donor DNA were carried on the same plasmid. This strategy yielded the deletion plasmid pEASY-T3-sgRNA-*melA*-*melA*^−^ for *melA* knockout and the integration plasmid pEASY-T3-sgRNA-*melA*-J23100-*cscB* for knock-in of the J23100–*cscB* expression cassette at the *melA* locus.

For genome editing, the 300 plasmid was first transformed into *E. coli* W, and transformants were selected on LB agar plates containing kanamycin at 30 °C to obtain a host strain stably carrying the Cas9/λ-Red module. The editing plasmids pEASY-T3-sgRNA-*melA*-*melA*^−^ or pEASY-T3-sgRNA-*melA*-J23100-*cscB* were then transformed into the 300-plasmid-positive strain and plated on LB agar containing ampicillin, kanamycin, and L-arabinose (2 g/L). Plates were incubated at 30 °C for 24–36 h. L-arabinose induced araC-regulated expression of Cas9 and the λ-Red recombination proteins (Gam, Bet, Exo), enabling Cas9 to introduce a double-strand break at the gRNA-specified target site and promoting homologous recombination between the chromosomal *melA* locus and the donor DNA carried on the editing plasmid.

Individual colonies were inoculated into LB broth containing ampicillin, kanamycin, and L-arabinose (2 g/L) and cultivated at 30 °C and 200 rpm for 12–16 h to further enhance editing efficiency. Colony PCR or genomic PCR was then performed using primers flanking the *melA* locus to detect changes in fragment size, and the resulting PCR products were sequenced to confirm successful *melA* deletion or J23100–*cscB* integration.

Verified positive clones were subsequently cultured in antibiotic-free LB medium at 37 °C for 3–5 successive passages to promote spontaneous loss of both the 300 plasmid and the pEASY-T3-based editing plasmids. Clones sensitive to ampicillin and kanamycin were selected and re-examined by PCR to confirm retention of the desired chromosomal modifications but loss of the editing plasmids. The resulting strains were designated *E. coli* W *melA*^−^ and *E. coli* W *melA*^−^ *cscB*^+^.

### 2.4. Soybean Molasses Substrate and Preparation

Soybean molasses used in this study was provided by Xuzhou Mutian Agricultural Technology Co., Ltd. (Xuzhou, China) as a by-product of the ethanol precipitation process for producing soy protein concentrate (SPC). Upon arrival, soybean molasses was stored at 4 °C and mixed thoroughly before use.

For fermentation experiments, soybean molasses was diluted with sterile distilled water to the desired soluble solids content (2–8 °Brix). The soluble solids content of the medium was measured using a digital refractometer (PAL-1, ATAGO, Tokyo, Japan) and adjusted to the target value prior to sterilization. For each culture, 50 mL of diluted soybean molasses solution was dispensed into a 150 mL Erlenmeyer flask, sealed, and sterilized at 121 °C for 20 min. After autoclaving, the media were cooled to room temperature and used as soybean molasses fermentation medium.

### 2.5. Media and Culture Conditions

#### 2.5.1. Media

Luria–Bertani (LB) medium was used for strain activation and routine molecular biology procedures. LB broth contained (per liter): NaCl, 10 g; yeast extract, 5 g; and tryptone, 10 g, dissolved in deionized water. LB agar plates were prepared by adding agar (18 g/L) to LB broth. For each culture, 50 mL of diluted molasses was dispensed into a 150 mL Erlenmeyer flask, capped with breathable closures, and sterilized at 121 °C for 20 min. Antibiotics and L-arabinose supplementation. Unless otherwise specified, ampicillin and kanamycin were added to final concentrations of 50 μg/mL when required. Antibiotics were prepared as sterile, filter-sterilized stock solutions (0.22 μm) and added aseptically to cooled media at 1:1000 (*v*/*v*). L-arabinose was prepared as a 200 g/L sterile stock solution and added to a final concentration of 2 g/L for induction of the araC-regulated promoter. In the single-factor and response surface optimization experiments, diammonium hydrogen phosphate ((NH4)_2_HPO_4_) was used as the nitrogen source.

#### 2.5.2. Culture Conditions

Culture conditions for molecular biology procedures. Molecular cloning and plasmid propagation were performed in LB medium. *E. coli* DH5α, *E. coli* W, and the engineered *E. coli* W strains were cultivated in shake flasks at 37 °C and 200 rpm, with antibiotics added as required (ampicillin, 50 μg/mL; kanamycin, 50 μg/mL).

Fermentation of *E. coli* strains in soybean molasses. *E. coli* W, *E. coli* BL21, and the engineered strains (*E. coli* W *melA*^−^ and *E. coli* W *melA*^−^ *cscB*^+^) were first streaked on LB agar plates and incubated at 37 °C for 14 h. Single colonies were then inoculated into 5 mL LB broth and pre-cultured at 37 °C and 200 rpm for 12–16 h. After pre-cultivation, the optical density at 600 nm (OD_600_) was measured, and the cultures were adjusted to OD_600_ ≈ 0.8 by dilution with fresh LB or by concentration. The cultures were inoculated into 150 mL Erlenmeyer flasks containing 50 mL soybean molasses fermentation medium at the desired inoculation ratio (*v*/*v*). Fermentations were carried out at 37 °C and 200 rpm for 72 h. Samples were collected every 12 h for analysis of sugar composition and cell growth.

Cultivation and fermentation of W34/70 and GS115 in soybean molasses. W34/70 and GS115 were pre-cultured in YPD medium (yeast extract, 10 g/L; peptone, 20 g/L; glucose, 20 g/L). The strains were streaked on YPD agar plates and incubated at 30 °C for 24–48 h. Single colonies were inoculated into 5 mL YPD broth and cultivated at 30 °C and 200 rpm for 16–18 h. After pre-cultivation, OD_600_ was measured and adjusted to approximately 0.8. The yeast cultures were inoculated into soybean molasses fermentation medium (50 mL per 150 mL Erlenmeyer flask) at the desired inoculation ratio (*v*/*v*), followed by fermentation at 30 °C and 200 rpm for 72 h. Samples were collected every 12 h for analysis of sugar composition and cell growth. All fermentation experiments were performed in at least triplicate, and the results are presented as mean ± standard deviation.

### 2.6. Analytical Methods

#### 2.6.1. Sample Preparation

For extracellular analysis, 1 mL of fermentation broth was transferred to a microcentrifuge tube and centrifuged at 11,000× *g* for 15 min. The resulting supernatant was collected as the extracellular sample. For whole-broth analysis, 1 mL of fermentation broth was transferred to a microcentrifuge tube, boiled at 100 °C for 8 min, and then centrifuged at 11,000× *g* for 15 min. The supernatant was collected as the whole-broth sample, containing both extracellular and heat-released intracellular components.

All samples were passed through 0.22 μm membrane filters prior to high-performance liquid chromatography (HPLC) analysis.

#### 2.6.2. HPLC Analysis of Stachyose, Sucrose, Glucose, Fructose and Related Sugars

Stachyose, sucrose, glucose, fructose, and other soluble sugars were quantified by HPLC. The HPLC system consisted of a Shimadzu (Kyoto, Japan) chromatograph equipped with a refractive index detector (RID, model RID-20A, Shimadzu, Kyoto, Japan). Separation was performed on an amino-bonded silica column packed with Hypersil NH2-S (4.6 × 250 mm, 5 μm; Dalian Elite Analytical Instruments Co., Ltd., Dalian, China). The mobile phase was acetonitrile–water (75:25, *v*/*v*) at a flow rate of 1.0 mL/min. The column temperature was maintained at 40 °C, and the injection volume was 10 μL. Prior to injection, all samples were filtered through 0.22 μm membrane filters to remove particulates and ensure sterility. Stachyose, raffinose, sucrose, glucose, and fructose standards were purchased from Solarbio (Beijing Solarbio Science & Technology Co., Ltd., Beijing, China), and quantification was performed using calibration curves established with the corresponding authentic standards. Peak assignment in soybean molasses samples was performed based on retention behavior under the same chromatographic conditions together with authentic standards for the major quantified sugars, and the resolved peaks were integrated separately for quantification.

In addition to absolute concentrations, relative concentrations of individual sugars were calculated based on peak area ratios. For each sampling point, the relative concentration was defined as the peak area of a given sugar at that time divided by its peak area in the initial fermentation sample (before inoculation).

#### 2.6.3. Measurement of Cell Growth (OD_600_)

Cell growth was monitored by measuring the optical density at 600 nm (OD_600_) using a SpectraMax i3 spectrophotometer (Molecular Devices, San Jose, CA, USA). An appropriate volume of fermentation broth was withdrawn and diluted with sterile medium when necessary to obtain OD_600_ values within the linear range (0.1–0.8). Non-inoculated medium was used as the blank. The absorbance at 600 nm was recorded, and the actual OD_600_ values were calculated by multiplying by the corresponding dilution factor.

## 3. Results

### 3.1. Types and Content of Sugars in Soy Molasses

**Figure 1:**
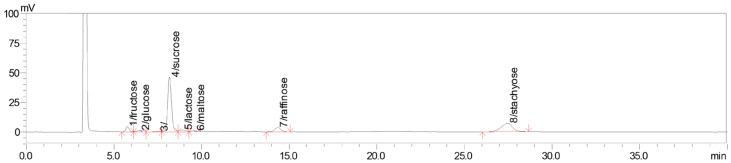
High-performance liquid chromatogram (HPLC) of soybean molasses supernatant at 2 °Brix. The detected peaks were assigned based on retention behavior under the same chromatographic conditions together with authentic standards for the major quantified sugars, and the corresponding peaks for fructose, glucose, sucrose, lactose, maltose, raffinose, and stachyose are indicated.

To evaluate the feasibility of selective stachyose enrichment, the identities and concentrations of individual sugars in soybean molasses were first determined to identify the major non-target sugars present in the substrate. In this study, stachyose purity on a TSS basis was used as a composition-based enrichment index derived from the major soluble sugars quantified by high-performance liquid chromatography (HPLC), rather than as an absolute purity value based on all soluble fermentation products. As shown in Table 3 and Figure 1, sucrose was the predominant sugar component in soybean molasses (53.20%, *w*/*w*), followed by stachyose (26.24%, *w*/*w*) and raffinose (6.76%, *w*/*w*), indicating a sucrose-dominant carbohydrate profile.

These results showed that soybean molasses contained a high proportion of sucrose together with a substantial amount of stachyose, suggesting that selective depletion of non-target sugars could provide a feasible route for stachyose enrichment.

### 3.2. Sucrose Consumption and Stachyose Retention Profiles of Different Chassis Strains in Soybean Molasses

**Figure 2:**
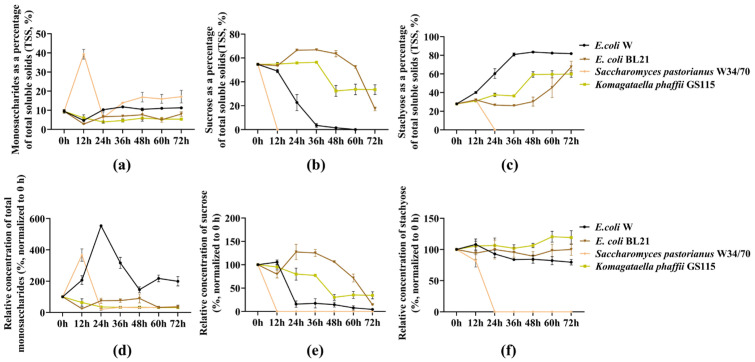
Temporal changes in soluble sugar composition and relative sugar levels in soybean molasses fermented by different strains. (**a**) Monosaccharides expressed as percentages of total soluble solids (TSS); (**b**) sucrose expressed as a percentage of TSS; (**c**) stachyose expressed as a percentage of TSS; (**d**) relative concentration of total monosaccharides (%, normalized to 0 h); (**e**) relative concentration of sucrose (%, normalized to 0 h); (**f**) relative concentration of stachyose (%, normalized to 0 h). The strains tested were *E. coli* W, *E. coli* BL21, W34/70, and GS115, as indicated. Data are shown as mean ± s.d.

To identify a suitable chassis for selective stachyose enrichment, four commonly used microbial strains, Escherichia coli W, *E. coli* BL21, W34/70, and GS115, were compared in terms of their fermentation behavior and sugar utilization patterns in soybean molasses. During fermentation, samples were collected every 12 h, and sugar concentrations were quantified by HPLC. The temporal changes in monosaccharides, sucrose, and stachyose are shown in Figure 2.

At 0 h, monosaccharides accounted for only a small fraction of the total soluble solids (TSS) in soybean molasses (Figure 2a), whereas sucrose and stachyose dominated the sugar composition (Figure 2b,c). Distinct strain-dependent differences were observed in the consumption of sucrose and stachyose during fermentation. In *E. coli* W, sucrose was rapidly depleted between 12 and 36 h: sucrose as a percentage of TSS decreased sharply from 54.64% to 3.50% (Figure 2b), and its relative concentration (normalized to 0 h) decreased in parallel and remained low thereafter (Figure 2e). Throughout fermentation, monosaccharides remained low in both their percentage of TSS and their relative concentration, showing only minor fluctuations (Figure 2a,d). In contrast, stachyose showed marked enrichment in composition; the proportion of stachyose increased continuously from 27.85% to 81.79% within 0–36 h and then remained at a high level (Figure 2c), while its relative concentration showed only a slight decline (Figure 2f). These results indicated that *E. coli* W preferentially consumed sucrose while retaining most of the stachyose.

In *E. coli* BL21, sucrose consumption was markedly slower. Sucrose as a percentage of TSS remained above 50.00% during 0–48 h and stayed at a relatively high level; during 48–72 h, it decreased rapidly from 63.57% to 17.37% (Figure 2b), accompanied by a corresponding decline in its relative concentration (Figure 2e). Stachyose accumulated in composition during 48–72 h, coinciding with the rapid depletion of sucrose (Figure 2c), whereas its relative concentration remained comparatively stable overall (Figure 2f). These results indicated that *E. coli* BL21 showed limited sucrose utilization during the early stage of fermentation.

In W34/70, sucrose was exhausted within 12 h, decreasing from 54.63% to 0.00% of TSS (Figure 2b), with a concomitant collapse in its relative concentration (Figure 2e). A pronounced transient increase in monosaccharides was observed at 12 h (Figure 2a,d), followed by a rapid decline. However, W34/70 also showed strong oligosaccharide utilization and nearly completely removed stachyose within 24 h in both composition and relative concentration (Figure 2c,f), indicating that this strain was unsuitable for the goal of preserving stachyose.

By contrast, GS115 began to rapidly utilize sucrose only at 36–48 h. During 48–72 h, both the proportion and relative concentration of sucrose approached a plateau, yet more than 30% sucrose remained (Figure 2b,e). Meanwhile, the proportion of stachyose increased over time, showing rapid accumulation during 36–48 h before stabilizing thereafter (Figure 2c), while its relative concentration remained stable or slightly increased at later time points (Figure 2f).

Overall, among the four strains evaluated, W34/70 was excluded because it almost completely consumed stachyose, whereas the other three strains achieved stachyose enrichment to different extents. Notably, *E. coli* W exhibited the fastest sucrose depletion and achieved the highest proportion of stachyose in TSS (80.84%). Although BL21 and GS115 did not show pronounced stachyose loss, both displayed lower enrichment efficiency than *E. coli* W. Therefore, *E. coli* W was selected as the chassis strain for subsequent genetic engineering.

### 3.3. Construction and PCR Verification of Engineered Strains with melA Deletion and cscB Overexpression

**Figure 3:**
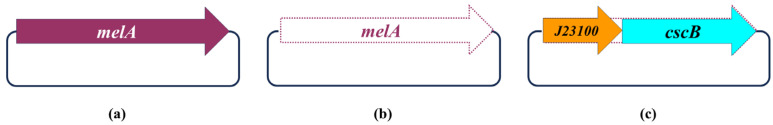
Schematic representation of the genomic configurations of the parental and engineered strains. (**a**) Parental *E. coli* W. (**b**) *E. coli* W *melA*^−^. (**c**) *E. coli* W *melA^−^ cscB^+^*, with the J23100–*cscB* cassette integrated at the *melA* locus.

To enable efficient sucrose consumption while suppressing stachyose hydrolysis, a targeted metabolic-engineering strategy was designed in *E. coli* W. First, the endogenous α-galactosidase-encoding gene *melA* was deleted to reduce stachyose hydrolysis. Second, a *cscB* overexpression strain was constructed on the *melA*^−^ background to further enhance sucrose uptake and consumption. Based on this design, two genome-editing plasmids were constructed: pEASY-T3-sgRNA-*melA*-*melA*^−^ for *melA* deletion and pEASY-T3-sgRNA-*melA*-J23100–*cscB* for integration of the J23100-driven *cscB* expression cassette (Figure 3).

**Figure 4:**
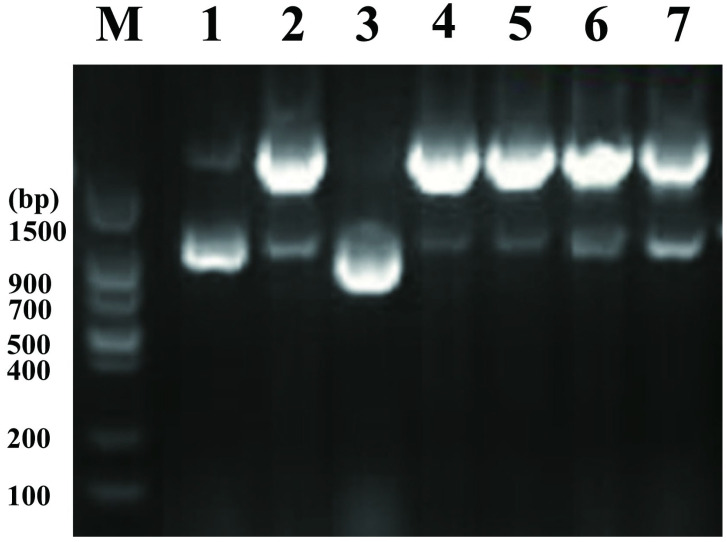
PCR genotyping of *melA* deletion in recombinant strains. Genomic DNA was amplified using primers F-*melA*-upout-c and R-*melA*-down-c flanking the *melA* locus. The expected amplicon sizes were 2308 bp for the parental non-deleted allele and 952 bp for the *melA*^−^ allele. Lane M, *Trans* DNA Marker II; lanes 1–7, independently screened recombinant colonies/strains. A 952 bp product was observed in lane 3, consistent with complete deletion of *melA*, whereas lanes 1, 2 and 4–7 yielded the 2308 bp product, indicating unsuccessful *melA* deletion in these strains.

PCR genotyping of *E. coli* W *melA*^−^ is shown in Figure 4. Genomic DNA was amplified using primers F-*melA*-upout-c and R-*melA*-down-c flanking the *melA* locus. The expected amplicon sizes were 2308 bp for the parental (non-deleted) allele and 952 bp for the *melA*^−^ allele. Among the independently screened recombinant colonies (lanes 1–7), a 952 bp product was observed in lane 3, consistent with successful deletion of *melA*, whereas lanes 1, 2, and 4–7 yielded the 2308 bp product, indicating that *melA* deletion had not occurred in these colonies.

**Figure 5:**
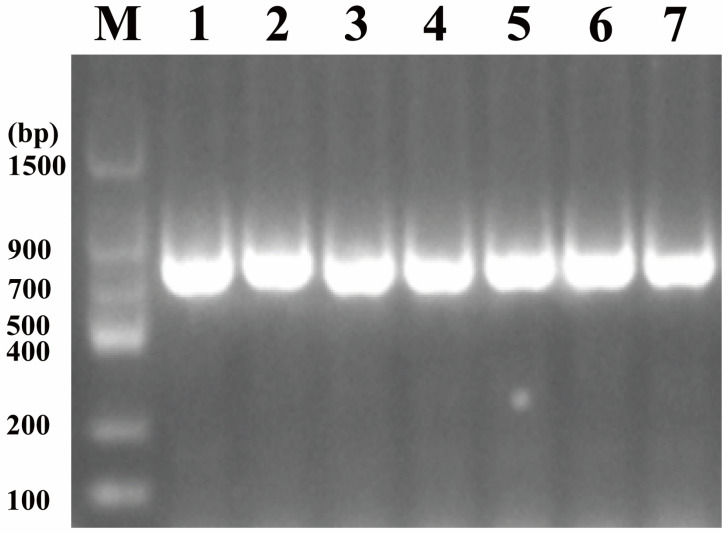
PCR verification of J23100–*cscB* knock-in using gene-specific primers. Genomic DNA was amplified using primers F-*cscB*-upout-c and R-J23100-*cscB*-c to detect the correct integration of the J23100–*cscB* cassette. The expected amplicon size for successful knock-in was 799 bp. Lane M, *Trans* DNA Marker II; lanes 1–7, the same colonies/strains as in Figure 4. All tested strains (lanes 1–7) produced the 799 bp product, confirming successful integration of J23100–*cscB*.

PCR verification of *E. coli* W *melA*^−^ *cscB*^+^ is shown in Figure 5. Genomic DNA was amplified using primers F-*cscB*-upout-c and R-J23100-*cscB*-c to detect correct integration of the J23100–*cscB* cassette. The expected amplicon size for successful knock-in was 799 bp. All tested colonies (lanes 1–7) produced the expected 799 bp product, confirming successful integration of the J23100–*cscB* cassette. These results confirmed the successful construction of the two engineered strains, *E. coli* W *melA^−^* and *E. coli* W *melA^−^ cscB^+^*.

### 3.4. Selective Retention and Enrichment of Stachyose in Soybean Molasses Enabled by melA Deletion and cscB Overexpression

**Figure 6:**
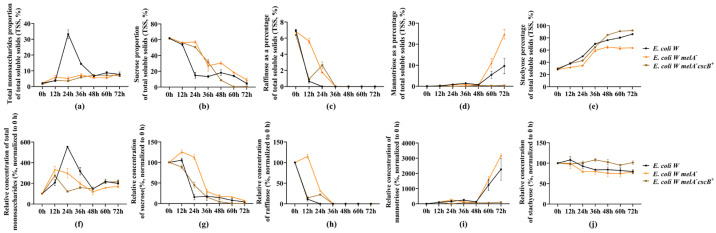
Time-resolved changes in soluble sugar profiles during cultivation of *E. coli* W and its engineered derivatives. (**a**–**e**) Sugar composition expressed as a percentage of total soluble solids (TSS): monosaccharides (**a**), sucrose (**b**), raffinose (**c**), mannotriose (**d**), and stachyose (**e**). (**f**–**j**) Relative concentrations (%, normalized to 0 h) of monosaccharides (**f**), sucrose (**g**), raffinose (**h**), mannotriose (**i**), and stachyose (**j**). Black, *E. coli* W; orange, *E. coli* W *melA*^−^; brown, *E. coli* W *melA*^−^ *cscB*^+^. Points represent means and error bars indicate s.d.

After genome editing, soybean molasses was used as the substrate to compare the 72 h fermentation performance of *E. coli* W, *E. coli* W *melA*^−^, and *E. coli* W *melA*^−^ *cscB*^+^, while tracking time-resolved changes in soluble sugar profiles (Figure 6). Panels a–e show sugar composition expressed as a percentage of total soluble solids (TSS), whereas panels f–j show the relative concentration of each sugar (%, normalized to 0 h).

The three strains exhibited markedly different utilization patterns for sucrose, raffinose, and stachyose. First, all strains showed pronounced sucrose consumption during fermentation (Figure 6b,g). At 0 h, sucrose accounted for approximately 60% of TSS and decreased rapidly within the first 24 h. The parental *E. coli* W showed the greatest relative sucrose depletion during 12–24 h, accompanied by a transient increase in monosaccharides (Figure 6a,f). In contrast, *E. coli* W *melA*^−^ *cscB*^+^ consumed sucrose slightly more slowly than the parental strain during the first 24 h, but maintained strong sucrose utilization after 48 h and reduced residual sucrose to below 1% by 72 h. By comparison, both the parental strain and *melA*^−^ strains still showed detectable sucrose at 72 h (Figure 6b,g).

Raffinose was rapidly depleted by all three strains (Figure 6c,h). Initially, raffinose accounted for approximately 6% of TSS. During 0–12 h, raffinose decreased rapidly to about 1% in the parental *E. coli* W and *E. coli* W *melA*^−^ *cscB*^+^, whereas the decline in *E. coli* W *melA*^−^ was comparatively slower. During 12–24 h, the parental strain further depleted raffinose to below the detection limit, and raffinose in *E. coli* W *melA*^−^ also dropped rapidly and became undetectable before 36 h. In *E. coli* W *melA*^−^ *cscB*^+^, raffinose showed a slight transient increase before being depleted to below detection.

Compared with sucrose and raffinose, the strain-dependent differences were more pronounced for stachyose utilization (Figure 6e,j). At the start of fermentation, stachyose accounted for approximately 27% of TSS. In the parental and *melA*^−^ strains, stachyose decreased to varying extents over time, accompanied by a marked increase in mannotriose (Figure 6d,i), with the highest mannotriose accumulation observed in *melA*^−^. In contrast, *E. coli* W *melA*^−^ *cscB*^+^ showed minimal stachyose consumption throughout fermentation: the proportion of stachyose increased from 29.95% to approximately 92.08%, and no obvious mannotriose accumulation was detected (Figure 6d,e,i,j).

Changes in monosaccharides further reflected the dynamics of sugar transformation during fermentation (Figure 6a,f). As sucrose and raffinose were depleted, all three strains exhibited a transient rise in monosaccharides during 12–24 h, with the highest peak observed in the parental *E. coli* W. Monosaccharide levels subsequently declined, indicating rapid assimilation during continued fermentation.

Under the tested conditions, *E. coli* W *melA^−^ cscB^+^* removed sucrose and raffinose from soybean molasses within 72 h while retaining most of the stachyose, which increased the stachyose fraction of total soluble solids (TSS) and prevented mannotriose accumulation. These results indicate that the engineered strain achieved selective removal of non-target sugars with concurrent enrichment of stachyose.

### 3.5. Process Parameter Screening and Response Surface Optimization for Stachyose Purity

**Figure 7:**
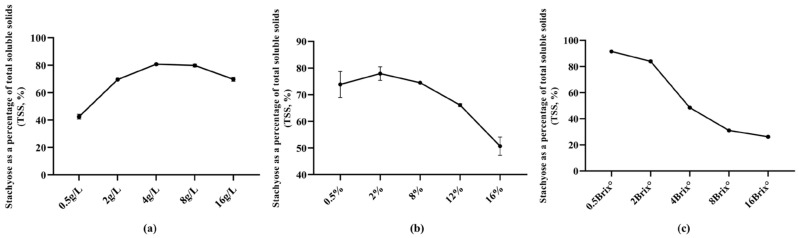
Single-factor effects on stachyose purity. (**a**) Nitrogen source level (g/L); (**b**) inoculum size (%); (**c**) initial soluble solids content (°Brix). The response is expressed as stachyose as a percentage of total soluble solids (TSS, %). Points represent means, and error bars indicate s.d. where shown.

To identify key process parameters affecting stachyose purity and define suitable factor ranges for response surface optimization, stachyose purity was used as the response variable, and the single-factor effects of nitrogen source level, inoculum size, and initial soluble solids content (°Brix) were evaluated. The results are shown in Figure 7a–c.

Nitrogen source level significantly affected stachyose purity (Figure 7a). Increasing the nitrogen source from 0.5 to 4 g/L raised the stachyose proportion from approximately 40% to 80%, indicating a clear positive trend. Further increasing the nitrogen source to 8–16 g/L led to a slight decline in stachyose purity, suggesting reduced selectivity under excessive nitrogen supply. Therefore, a nitrogen source range of 2–8 g/L was selected for subsequent optimization.

Inoculum size also influenced stachyose purity (Figure 7b). When inoculum size increased from 0.5% to 2%, stachyose purity increased slightly and reached a maximum at approximately 2%. Increasing inoculum size further to 8% caused little change, whereas stachyose purity decreased markedly at 12% and 16%. These results indicated that both excessively low and excessively high inoculum sizes were unfavorable for stachyose enrichment. Accordingly, an inoculum size range of 0.5–8% was selected for subsequent optimization.

Initial soluble solids content (°Brix) exerted the most pronounced effect on stachyose purity (Figure 7c). At low °Brix, the proportion of stachyose in TSS was the highest. As °Brix increased from 2 to 4 and 8 °Brix, stachyose purity decreased progressively, with a sharper decline at higher °Brix. Because °Brix showed the strongest effect on stachyose purity, it was selected as a key variable for subsequent optimization. Fermentation temperature and pH were treated as fixed parameters because they showed only minor effects on the response under the tested conditions.

In summary, the single-factor experiments indicated that nitrogen source level, inoculum size, and initial °Brix all influenced stachyose purity, with °Brix showing the strongest effect. Therefore, nitrogen source level (A), inoculum size (B), and initial °Brix (C) were selected as independent variables for Box–Behnken response surface optimization.

Using stachyose purity as the response (Y), a second-order polynomial regression model was established with nitrogen source level (A), inoculum size (B), and initial °Brix (C) as independent variables, with the corresponding actual factor levels shown in Table 4. The ANOVA results of the model are presented in Table 5. The overall model was highly significant (F = 84.53, *p* < 0.0001), and the lack-of-fit test was not significant (*p* = 0.4872), indicating good model adequacy. The model also showed a high coefficient of determination (R^2^ = 0.9909), with adjusted R^2^ (0.9792) and predicted R^2^ (0.9301) in reasonable agreement, indicating strong goodness-of-fit and predictive ability.

Regarding term significance, the linear effect of °Brix (C) on the response was highly significant (*p* < 0.0001), and the quadratic term C^2^ was also significant (*p* = 0.00026) (Table 5). These results indicate a pronounced quadratic response to °Brix, suggesting the presence of an optimal °Brix range. In contrast, the linear terms of nitrogen source level (A) and inoculum size (B), as well as their quadratic terms (A^2^ and B^2^), were not significant at the 0.05 level (*p* > 0.05), indicating comparatively weaker effects within the tested ranges. None of the interaction terms (AB, AC, and BC) reached statistical significance, suggesting minimal interaction among the factors. Overall, the Box–Behnken regression model captured the combined effects of nitrogen source level, inoculum size, and °Brix on stachyose purity, with °Brix identified as the most influential factor.

The fitted quadratic regression equation in terms of coded variables was as follows:

(1) Y = 51.68 + 2.02A − 1.06B − 27.41C − 1.08AB + 1.29AC + 2.66BC − 0.83A^2^ − 1.77B^2^ + 9.64C^2^

Using the fitted response surface model, the optimal conditions were predicted to be a nitrogen source (diammonium hydrogen phosphate) level of 5.9 g/L, an inoculum size of 1.5%, and an initial soluble solids content of 2 °Brix, yielding a predicted maximum stachyose purity of 90.85%. Under these conditions, a validation experiment produced a stachyose purity of 90.91 ± 0.52% at 60 h, in close agreement with the predicted value, confirming the accuracy of the model.

**Table 4:** Factors and coded levels used in the Box–Behnken design (BBD).

Level	Nitrogen Source (g/L)	Inoculum Size (%)	Initial °Brix of the Fermentation Broth (°Brix)
−1	2	0.5	2
0	5	4.25	4
+1	8	8	8

**Table 5:** Analysis of variance (ANOVA) for the response surface regression model of stachyose purity (TSS, %).

Source	Sum of Squares	df	Mean Square	F-Value	*p*-Value	Significance
Model	6491.18	9	721.24	84.5252	<0.0001	**
A (Nitrogen source)	32.55	1	32.55	3.8144	0.091756	
B (Inoculum size)	9.03	1	9.03	1.0580	0.337884	
C (°Brix)	6011.63	1	6011.63	704.5265	<0.0001	**
AB	4.67	1	4.67	0.5472	0.483544	
AC	6.66	1	6.66	0.7804	0.406319	
BC	28.23	1	28.23	3.3080	0.111768	
A^2^	2.89	1	2.89	0.3385	0.578923	
B^2^	13.21	1	13.21	1.5477	0.253516	
C^2^	391.03	1	391.03	45.8260	0.00026	**
Residual	59.73	7	8.53			
Lack of fit	25.25	3	8.42	0.9762	0.487236	ns
Pure error	34.48	4	8.62			
Total	6550.91	16				

Model fit statistics: R^2^ = 0.9909; Adjusted R^2^ = 0.9792; Predicted R^2^ = 0.9301. ** indicates *p* < 0.01; ns indicates not significant.

## 4. Discussion

### 4.1. Soybean Molasses Provides a Suitable Substrate for Selective Biological Enrichment of Stachyose

In this study, soybean molasses was confirmed to be a suitable substrate for selective stachyose enrichment because sucrose was the predominant soluble sugar, while stachyose was also present at a relatively high proportion. This compositional feature is important for process design. On the one hand, the high sucrose content increases the difficulty of downstream purification because it contributes substantially to the background sugar pool. On the other hand, the substantial native abundance of stachyose means that selective removal of non-target sugars can markedly increase the relative proportion of the target oligosaccharide without requiring de novo biosynthesis. Therefore, soybean molasses is not only a low-cost by-product, but also a structurally favorable substrate for biological pre-purification of stachyose.

These findings are consistent with previous reports describing soybean molasses as a carbohydrate-rich by-product dominated by sucrose and raffinose family oligosaccharides. However, our results further emphasize that the value of soybean molasses lies not merely in its use as a general fermentation feedstock, but in its suitability for selective sugar removal and target oligosaccharide enrichment. In this sense, the present work extends the concept of by-product valorization from bulk substrate utilization to selective compositional upgrading. This distinction is particularly relevant for functional carbohydrate production, where relative purity is often as important as absolute yield.

### 4.2. Chassis-Dependent Sugar Utilization Is a Key Determinant of Stachyose Enrichment Selectivity

A major finding of this study is that the choice of microbial chassis strongly determined the selectivity of stachyose enrichment in soybean molasses. Although all tested strains were able to consume soluble sugars to varying extents, they differed substantially in the balance between sucrose depletion and stachyose preservation. Among the four strains examined, *E. coli* W exhibited the most favorable phenotype, characterized by rapid sucrose removal together with limited stachyose consumption [16,24]. By contrast, W34/70 removed sucrose very efficiently but also consumed stachyose almost completely, making it unsuitable for a purification strategy aimed at retaining the target oligosaccharide [33]. BL21 and GS115 showed relatively limited direct loss of stachyose, but their sucrose utilization rates were lower than that of *E. coli* W, resulting in slower and less efficient enrichment [19,34].

These results suggest that selective biological purification cannot be achieved simply by choosing a strain that grows well in molasses or that consumes sugars rapidly. Rather, the intrinsic hierarchy of substrate utilization and the sugar transport and hydrolysis characteristics of the host are critical determinants of process selectivity [16]. Because sucrose is the predominant non-target sugar in soybean molasses, the initial chassis-screening step in this study was designed primarily to identify strains with favorable sucrose and non-target sugar consumption behavior while minimizing stachyose loss. In particular, the superior performance of *E. coli* W in the present study is more likely attributable to its efficient baseline sucrose utilization behavior, supported by the native csc pathway, than to a confirmed strain-specific transport advantage for higher-order oligosaccharides This interpretation is consistent with previous studies showing that mixed-carbon fermentation behavior is highly strain-dependent and that the microbial preference for specific carbohydrates can profoundly affect product composition. In the context of soybean molasses, an ideal chassis should not only remove sucrose efficiently, but should also avoid substantial utilization of stachyose. Our results indicate that *E. coli* W best satisfies this dual requirement and therefore provides a rational starting point for targeted engineering [24].

### 4.3. Combined melA Deletion and cscB Overexpression Improve Selective Retention of Stachyose

The engineering results further demonstrate that selective stachyose biopurification depends on coordinated regulation of both target-sugar protection and non-target-sugar consumption. Deletion of *melA* altered the dynamics of stachyose-related sugar conversion, indicating that the endogenous α-galactosidase pathway contributes to target-sugar loss in the parental strain. However, *melA* deletion alone was insufficient to completely prevent stachyose decline, and it was accompanied by marked accumulation of mannotriose. This result suggests that, although *melA* is involved in stachyose conversion, additional routes for oligosaccharide transformation likely remain active in the system [16].

By contrast, integration of the J23100-driven *cscB* cassette into the *melA*^−^ background produced a much more favorable phenotype. The resulting strain, *E. coli* W *melA*^−^ *cscB*^+^, not only maintained strong sucrose utilization during the later phase of fermentation, but also showed minimal stachyose consumption and no obvious accumulation of mannotriose. These results indicate that overexpression of *cscB* did more than accelerate sucrose metabolism in the early stage; more importantly, it appeared to maintain sucrose uptake capacity in the later stage of fermentation [16], when easily assimilable carbon sources became limited. Under such conditions, insufficient sucrose uptake may increase the likelihood that cells shift toward alternative oligosaccharide conversion routes. This pattern suggests that the observed phenotype may be related to a kinetic or flux-based effect, in which sustained sucrose uptake and utilization reduce the need for alternative oligosaccharide conversion, rather than to active repression of other enzymes. Enhancing sucrose transport therefore seems to reduce the selective pressure for stachyose utilization and indirectly protects the target sugar [16].

From a process-engineering perspective, this result is important because it shows that blocking target-sugar degradation alone is not enough. Efficient and sustained removal of the competing non-target sugar pool is equally necessary. In other words, selective biopurification in complex natural sugar systems should be understood as a dual-objective engineering problem involving both preservation of the desired oligosaccharide and preferential depletion of background sugars. The success of the *melA*^−^ *cscB*^+^ strategy provides proof of concept that this dual-objective design can substantially improve purification selectivity.

At the same time, the observed accumulation of mannotriose in the parental and *melA*^−^ strains suggests that the conversion network of soybean oligosaccharides in *E. coli* W is not yet fully resolved. The present results support a working hypothesis that alternative fructosyl-end cleavage or related side-conversion routes may contribute to stachyose loss when sucrose uptake becomes limiting. Based on the observed conversion pattern and KEGG pathway analysis, a plausible candidate is β-fructofuranosidase activity (EC 3.2.1.26), which can cleave the terminal fructosyl moiety of stachyose to generate mannotriose and fructose; however, the responsible enzyme(s) were not directly identified in the present study. Future studies combining transcriptomics, metabolite profiling, and enzyme activity assays would be valuable for identifying the enzymes and regulatory nodes involved.

### 4.4. Process Optimization Further Improves Stachyose Purity and Supports Future Scale-Up and Valorization

In addition to strain engineering, fermentation conditions played a major role in determining the final enrichment outcome. Single-factor experiments and response surface analysis showed that nitrogen source level, inoculum size, and initial °Brix all affected stachyose purity, with initial °Brix being the most influential factor. The slight decline in stachyose purity observed at higher nitrogen levels may reflect enhanced biomass formation and increased overall carbon demand under excessive nitrogen supply, thereby reducing the selectivity of sugar consumption. Since diammonium hydrogen phosphate was used as the nitrogen source, strong pH-driven effects were not considered the primary explanation, although pH was not systematically monitored in the present study. These observations indicate that the metabolic advantages of the engineered strain can only be fully realized under an appropriate process environment. In practice, substrate loading, nutrient supply, and inoculation level jointly determine the extent to which the system can achieve rapid removal of non-target sugars while minimizing target-sugar loss.

Among the tested variables, °Brix had the strongest and most significant effect, and both its linear and quadratic terms were significant in the regression model. This result suggests that there is an optimal substrate concentration window for selective biopurification. At excessively high soluble solids concentrations, the efficiency of non-target sugar removal decreased and stachyose purity declined, indicating that higher substrate loading does not necessarily improve enrichment performance. By optimizing the selected variables using the Box–Behnken design [35], the predicted maximum stachyose purity exceeded 90%, and the validation experiment was in close agreement with the model prediction. This confirms that process optimization can further enhance the performance of the engineered strain and that the established model has practical value for defining an operational window. At the same time, the non-significant interaction terms suggest that, within the tested design space, the main factors act with limited mutual interference; however, this should not be interpreted to mean that scale-up can be achieved by controlling °Brix alone, because additional process variables may become important at larger scale.

Despite these promising results, several limitations should be noted. First, the present study was performed mainly at laboratory scale, and the fermentation system has not yet been validated under pilot-scale conditions. In larger-scale processes, batch-to-batch variation in soybean molasses composition, mass-transfer characteristics, and downstream integration may all affect product quality and process stability [36,37]. Although soybean molasses contains phytochemicals such as saponins and isoflavones, the engineered strain was still able to grow and carry out efficient non-target sugar removal under the tested conditions, suggesting that these components did not exert an obvious inhibitory effect on the biopurification process in the present system. However, their specific effects on strain growth and metabolic performance were not systematically evaluated against defined pure-sugar media and will require further study. Second, although the current engineering strategy successfully improved selectivity, the molecular basis of residual stachyose conversion remains incompletely understood. Third, the current process still treats sucrose consumption mainly as a means of impurity removal. In future work, it may be possible to redirect sucrose-derived carbon flux toward rapidly formed, metabolically compatible co-products, thereby coupling stachyose enrichment with carbon valorization while remaining compatible with the current fermentation timeline.

In this study, we established an integrated strategy for selective stachyose biopurification from soybean molasses by combining chassis selection, pathway-level engineering, and process optimization. Beyond this specific system, the work also highlights a broader principle for engineering complex carbohydrate fermentation processes: improving purification selectivity requires simultaneous consideration of target preservation, background sugar removal, and process-level operating conditions [38]. Further mechanistic studies and scale-up validation will be necessary to evaluate the industrial potential of this approach.

## 5. Conclusions

In this study, soybean molasses was successfully used as a substrate for the selective biological enrichment of stachyose through an integrated strategy combining chassis screening, CRISPR–Cas9-based metabolic engineering, and fermentation process optimization. Among the four strains evaluated, *Escherichia coli* W showed the best balance between rapid sucrose depletion and stachyose retention and was therefore selected as the chassis for further engineering. Deletion of *melA* reduced stachyose loss, whereas additional overexpression of *cscB* on the *melA*^−^ background further improved sucrose utilization and minimized stachyose degradation, resulting in a marked increase in stachyose purity from below 30% to above 80% (TSS basis). Subsequent response surface optimization identified initial °Brix as the most influential process factor, and under the optimized conditions, the engineered strain achieved a stachyose purity of 90.91 ± 0.52% at 60 h. These results indicate that selective preservation of stachyose can be achieved by coordinating target-sugar protection with efficient removal of non-target sugars. This work provides a practical strategy for stachyose enrichment from soybean molasses and offers a useful framework for the selective biopurification of raffinose family oligosaccharides from plant-derived by-products.

## Figures and Tables

**Table 1:** Strains and plasmids used in this study.

Strain or Plasmid	Relevant Characteristics	Source
Strains		
*E. coli* DH5α	Chemically competent cloning host for plasmid propagation	Purchased strain, maintained in our laboratory
*E. coli* W ATCC (9637)	Parental chassis strain	Strain maintained in our laboratory
*E. coli* BL21	Laboratory expression strain	Strain maintained in our laboratory
GS115	Laboratory *K. phaffii* host strain	Strain maintained in our laboratory
W34/70	Industrial lager-brewing strain	Strain maintained in our laboratory
*E. coli* W *melA*^−^	Engineered derivative of *E. coli* W with *melA* deletion	This study
*E. coli* W *melA*^−^ *cscB*^+^	Engineered *E. coli* W *melA*^−^ strain carrying a J23100–*cscB* expression cassette integrated at the *melA* locus	This study
Plasmids		
pRed_cas9_recA_Δpoxb300	Kan^R^; Cas9; araC; Gam, Bet, Exo recombination functions; repA101	Laboratory stock
pEASY-T3	Amp^R^ cloning vector	pEASY^®^-T3 Cloning Kit (TransGen Biotech)
pEASY-T3-sgRNA	Amp^R^; sgRNA expression vector	This study
pEASY-T3-sgRNA-*melA*	Amp^R^; *melA*-targeting sgRNA (sgRNA-*melA*)	This study
pEASY-T3-sgRNA-*melA*-J23100-*cscB*	Amp^R^; sgRNA-*melA*; J23100–*cscB* expression cassette flanked by *melA* homology arms (integrative editing plasmid)	This study

**Table 2:** Primers used in this study.

Primer	Sequence (5′–3′)
F-gRNA-*melA*	TTGACAGCTAGCTCAGTCCTAGGTATAATGCTAGCCCAACAGAAAGAAGCCTTACGTTTTAGAGCTAGAAATAGCAAGTT
R-gRNA-*melA*	GGTTCTTATGGCTCTTGTATCTATCAGTGAAGCATCAAGAC
F-*melA* -T3 -up	CACTAGTGAATTCGCGGCCGCCTGCAGCTGTGTGGCCAGTGATTTGATCACCAT
R-*melA*-down-up	GTAGCGTTTAGTCGCGTTGCAGATCTCCTGGCTTGCTTGAATAACTTCAT
F-*melA*-up-down	CAAGCCAGGAGATCTGCAACGCGACTAAACGCTACTGCGCCGGGGGAAT
R-*melA*-T3-down	CTCAAGCTATGCATCCAACGCGTTGGGGCGATTAGTACCAGAGTGAACATCTGAAAGC
F-down-T3-loop	CAAATCACTGGCCACACAGCTGCAGGCGGCCGCGAATT
R-up-T3-loop	CACTCTGGTACTAATCGCCCCAACGCGTTGGATGCAT
F-J23100-*cscB*	GCTCAGTCCTAGGTACAGTGCTAGCTACTAGAGAAAGAGGAGAAATACTAGATGGCACTGAATATTCCATT
R-down-*cscB*-arm	GTAGCGTTTAGTCGCGTTCTATATTGCTGAAGGTACAGGCGT
F-uparm-J2300-arm	CAAGCCAGGAGATCTGCTTGACGGCTAGCTCAGTCCTAGGT
F-*melA*-upout-c	CAGAATCGGTTCCCAGCCAGAG
R-*melA*-down-c	CGAACGCTCCAAATCCATAACTGAG
F-*cscB*-upout-c	CGTTCGGACTGTTTAATTCCTGCTG
R- J23100-*cscB*-c	GGAATATTCAGTGCCATCTAGTATTTCTCC

**Table 3:** Sugar composition of soybean molasses (%, *w*/*w*).

Sample	Sugar Components (%, *w*/*w*)						
	Fructose	Glucose	Sucrose	Lactose	Maltose	Raffinose	Stachyose
soybean molasses	4.44	1.44	53.20	2.86	1.33	6.76	26.24

Values are expressed as percentages of total detected soluble sugars.

## Data Availability

The original contributions presented in this study are included in the article. Further inquiries can be directed to the corresponding authors.

## Abbreviations

The following abbreviations are used in this manuscript:

RFOs	Raffinose family oligosaccharides
TSS	Total soluble solids
HPLC	High-performance liquid chromatography
RSM	Response surface methodology
BBD	Box–Behnken design
ANOVA	Analysis of variance

## Appendix A. Detailed PCR Reaction Mixtures

**Table A1:** PCR reaction mixture (50 μL).

Component	Volume (μL)
2× Rapid Taq Master Mix	25
Forward primer (10 μmol/L)	2
Reverse primer (10 μmol/L)	2
Template DNA (20 ng/μL)	1
Nuclease-free water	20
Total volume	50

**Table A2:** PCR reaction mixture (20 μL).

Component	Volume (μL)
2× Rapid Taq Master Mix	10
Forward primer (10 μmol/L)	0.5
Reverse primer (10 μmol/L)	0.5
Template DNA (20 ng/μL)	1
Nuclease-free water	8
Total volume	20
